# *Aequilibrium prudentis*: on the necessity for ethics and policy studies in the scientific and technological education of medical professionals

**DOI:** 10.1186/1472-6920-13-58

**Published:** 2013-04-23

**Authors:** Misti Ault Anderson, James Giordano

**Affiliations:** 1 Department of Microbiology and Immunology, Georgetown University Medical Center, 3900 Reservoir Road, Washington, DC 20057-1440, USA; 2 Potomac Institute for Policy Studies, 901 N. Stuart Street, Suite 200, Arlington, VA 22203, USA; 3 Neuroethics Studies Program, Edmund D. Pellegrino Center for Clinical Bioethics, and Division of Integrative Physiology, Department of Biochemistry, Georgetown University Medical Center, 4000 Reservoir Rd, Rm. 238, Bldg D, Washington, DC 20057, USA; 4 Human Science Center, Ludwig-Maximilians Universität, Goethestraße 31, Munich, D-80336, Germany; 5 Human Science Center, Ludwig-Maximilians Universität, Professor Max Lange Platz 11, Bad Tölz, 83646, Germany

**Keywords:** Medical education, STEM education, Ethics education, Global ethics, Biotechnology

## Abstract

**Background:**

The importance of strong science, technology, engineering, and mathematics education continues to grow as society, medicine, and the economy become increasingly focused and dependent upon bioscientific and technological innovation. New advances in frontier sciences (e.g., genetics, neuroscience, bio-engineering, nanoscience, cyberscience) generate ethical issues and questions regarding the use of novel technologies in medicine and public life.

**Discussion:**

In light of current emphasis upon science, technology, engineering, and mathematics education (at the pre-collegiate, undergraduate, graduate, and professional levels), the pace and extent of advancements in science and biotechnology, the increasingly technological orientation and capabilities of medicine, and the ways that medicine – as profession and practice – can engage such scientific and technological power upon the multi-cultural world-stage to affect the human predicament, human condition, and perhaps nature of the human being, we argue that it is critical that science, technology, engineering, and mathematics education go beyond technical understanding and directly address ethical, legal, social, and public policy implications of new innovations. Toward this end, we propose a paradigm of integrative science, technology, ethics, and policy studies that meets these needs through early and continued educational exposure that expands extant curricula of science, technology, engineering, and mathematics programs from the high school through collegiate, graduate, medical, and post-graduate medical education. We posit a synthetic approach that elucidates the historical, current, and potential interaction of scientific and biotechnological development in addition to the ethico-legal and social issues that are important to educate and sustain the next generation of medical and biomedical professionals who can appreciate, articulate, and address the realities of scientific and biotechnological progress given the shifting architectonics of the global social milieu.

**Summary:**

We assert that current trends in science, technology, medicine, and global politics dictate that these skills will be necessary to responsibly guide ethically sound employment of science, technology, and engineering advancements in medicine so as to enable more competent and humanitarian practice within an increasingly pluralistic world culture.

## Background

Scientific knowledge and technological tools have enabled the heuristics and advancements that have led to rapid progress in several fields (e.g., genomics, the neurosciences, bio-engineering, nanotechnology, cyberscience) that are constituent to the profession and practices of modern medicine. Indeed, much of the current scope, tenor, and capability of medicine have been expanded, if not defined, by scientific and technological capabilities [1]. Yet, medicine remains a deeply humanitarian discipline, characterized by its fundamental philosophical tenets of providing both (technically) right and (morally and ethically) good care to those rendered vulnerable by the predicament of injury, disease, and the infirmities of life [2]. However, the pace and profundity of such innovation and developments often exceed the extent, if not capability, of the ethical approaches and deliberation necessary both to address the issues that such biosciences and technology may generate, and to inform the guidelines, policies, and laws that guide and govern research and its applications in various domains within medicine (if not the social realm at large). Moreover, given the leverage that bioscience and technology confer and elicit upon an increasingly pluralist world stage, any such ethics, guidelines, and policies will need to be articulated in culturally sensitive ways, so as to best reflect the dynamics of what has been termed the contemporary “global shift” [3]. In light of this, we posit that education that is singularly focused upon either the sciences or the humanities will not be sufficiently deep or broad to prepare future practitioners and leaders to engage the challenges evoked by an ever-expanding capability of science and technology in medicine and/or society [4,5].

## Discussion

If we are to prioritize science, technology, engineering, and mathematics (i.e., STEM) pursuant to medical and biomedical education, training, and practice, then it is essential to do so in ways that genuinely appreciate and apprehend these pursuits as human enterprises that are devised and articulated, and impact the boundaries of human culture and its societies. In other words, we must ground STEM education to the reality that STEM – as contributory to medicine, and like medicine itself – are human endeavors for and within the sphere of humanity, human conduct, and human ecology. We opine that to meet the opportunities, benefits, challenges, and burdens generated by the study, development, and use of STEM in real-world scenarios, education must not only be multi-disciplinary, but must engage the natural, physical, and social sciences as well as the humanities in an integrative model that 1) obtains a solid foundation in humanitarian sensitivities, 2) engages knowledge of STEM to address the challenges of modern medicine enacted on a global scale, and 3) entails the humanities and social sciences to assess and direct the value and utility of STEM (as constituents of medical practice) within current culture, and as means to shape health, culture, and societies of the future.

Fortification of STEM education is important if we are to train medical and biomedical professionals to possess the knowledge, skills, and innovative acumen that will be required to keep pace with – and within – the worldwide groundswell of scientific and technological (S/T) progress. To be sure, S/T advancement requires significant economic support, and in turn, can elicit tremendous economic growth. Much of S/T research is conducted by, and subsidized through, the infrastructure of the so-called “triple helix” of academic, government, and commercial research institutions [6]. For example, a number of biotechnology companies have been developed and have evidenced sustained growth around prestigious universities (e.g., Silicon Valley near Stanford University; the venture capital enclave around Harvard, MIT, Northeastern, Tufts, and other academic institutions of Cambridge/Boston in the United States); these S/T nodes have been important to transferring knowledge from academic laboratories to the marketplace, and commercializing the outcomes, products, and tangible benefits of academic research in the corporate and public spheres including medicine. Moreover, such focal centers of triple helix activity are serving as a model for rapid S/T progress and are arising in other countries as well (e.g., the corporate-academic complexes of Munich and other cities in Germany; multiple sites in China and India, etc.). Thus, academic training in STEM has exerted, and will continue to exert, a direct effect upon industrial development and economic growth in the United States [6] and abroad.

On many levels, the triple helix model provides a viable framework for STEM education and training [7]. As hybrid fields develop through the strategic conjoinment of heretofore traditionally siloed scientific disciplines, advances spawned by integrative scientific convergence will both increase currently known and recognized technical, ethical, legal, and social challenges, and will foster other challenges that will be somewhat more unique given the novelty of methods, tools, outcomes, and effects [8]. To intuit, work, and succeed in the interdisciplinary environment of integrative scientific convergence within the triple helix, students and biomedical professionals must understand and be relatively fluent in epistemologies and human dynamics of academia, government, and industry. We assert that this will require a deeper and broader educational design that develops genuinely equivalent competencies in the sciences and humanities [7-9].

### Ethics and policy studies: “*In-STEP*” with STEM Education for – and in – medicine

Toward this end, we have proposed a model of integrative science, technology, ethics, and policy studies (viz.- *In-STEPS*) that provides a framework upon and through which to build competencies in the content, constructs, and applications of convergent sciences, as well as the knowledge and skill sets necessary to realistically observe, and meaningfully address and guide, the conduct and uses of science and technology as human enterprises [8,9]. The overarching goals of the *In-STEPS* paradigm are to integrate scientific and humanitarian disciplines at all levels from high school through undergraduate, graduate, medical and post-graduate medical education. This will 1) provide an early exposure to issues and social implications of scientific and technological research and applications; 2) generate a deep understanding of specific ethical, legal, and policy issues that are relevant to those ways that frontier domains of science and technology can be used or misused in medicine to alter the human condition, human predicament, and various aspects of social action; and 3) develop insights and capabilities to enable more thorough and valid deliberation and direction of the ways that science and technology should be employed within clinical care to sustain the values and goods of individuals, communities, and societies [8,9].

We maintain that integrating studies of ethical, legal, and social issues (ELSI) within the fabric of STEM-based pre-medical and medical education will be important to developing the abilities to responsibly (and arguably more effectively) work in and lead science, biotechnology, and medicine as well as those fields that inform and develop the policies and laws that steer and regulate science, technology, and medicine as socio-public resources, services, and good(s) [5,8,9]. In short, we argue that given the pace of S/T progress, it will be vital to develop a cadre of professionals that are literate in science and equally cognizant of the historicity of science and technology as elements that can strongly shape, and are at the same time vulnerable to the forces of culture. The requisite lens must view both humanity and science as being dynamic, rather than static or absolute [3], and in this way, afford perception that scientific knowledge – and medicine – are not immune to cultural beliefs, social norms, economics, and politics [10], p.267-8. Such literacy is required for the “effective communication of scientific and technological findings…necessary to make possible true public discussion of their ethical, legal and social implications” [11], p.438. Hence, integration of ELSI into STEM and medical education serves two goals: first, to engage non-science students to gain insight(s) to scientific information necessary to appreciate and analyze the social issues generated by the use of science and technology in medicine to address perdurable problems of human health and human potential; and second, to equip students in medicine and the biomedical sciences with the philosophical, historical, and ethical knowledge to foster insight to, and responsibility for, the ethical, legal, and social aspects and impact of their work [12].

Currently, both the United States’ National Science Foundation (NSF) and the National Institutes of Health (NIH) require training in the responsible conduct of research (RCR, i.e., some form of research ethics) for all students involved in projects and programs that are pursuing or have been awarded (NSF or NIH) grant funding [13,14]. Despite the NSF and NIH requirements for RCR in STEM education, a recent survey of undergraduate institutions revealed that only 37% of schools included an ethics component in the core curriculum; 13% of the schools indicated that biology majors were required to have (some) formal training in ethics, and only a third of those required an ethics of science course [15], indicating that training in research ethics has not spread beyond those immediately impacted by the mandate. It was also noted that the majority of instructors indicated that they spent less than 5% of lecture and discussion time, if any, addressing ethical and social issues in class [15]. Typical ethics courses and curricula in medical schools provide a basic foundation of concepts and clinical applications, and tend to function under one of two main goals: either creating virtuous physicians or giving physicians the skills and training to analyze and resolve ethico-legal dilemmas [16]. While these are reasonable goals, medical ethics training tends to fall short of providing a more fine-grained explication of those ways that science and technology can, have, and will affect medical research and practice, the ELSI that can be generated by this expanding capability and reliance upon S/T advances in medicine, and the knowledge and skill required to more genuinely and meaningfully address the potential and problems fostered by the power of this scientific and technological prowess to affect human health, wellness, and suffering in and across different cultures.

While NSF and NIH requirements stop short of mandating that institutions develop and provide full coursework to all students in S/T majors – inclusive of medicine – we maintain that such a dictate might in fact be defensible (and laudable). This would establish both a defined necessity for the purposeful conjoinment of ELSI-STEM, and do so as a *quid pro quo* such that ethics education would be required for potential and/or sustained funding of S/T research. It is possible that the addition of ethics coursework could be seen as a mere “check in the box” to secure ongoing fiscal support in the face of widespread university and federal budgetary cuts. Even so, this can still be seen as a plus-sum situation: if an adequate level of NSF and/or NIH funding were to be *provided* to support such ethics coursework (or curricula), appropriated monies could be used to re-fortify humanities’ programs that could be specifically designed to more authentically and effectively inter-digitate with the sciences and medicine.

However, while necessary, simply providing training in, or a course on RCR is not, to our view, in and of itself sufficient to meet either the full scope of NSF and NIH intent, or, more specifically the idea and agenda of *In-STEPS* as proposed herein. The importance of integrating ethics and policy into STEM education increases as S/T innovation outpaces the development of policy and law. To wit, the rate of scientific discovery and technological product development has increased over the past two decades. Using neuroscience and neurotechnology as an example, a mean increase of 73% across sub-disciplines (e.g., various forms of neuroimaging, neurogenetics, neuroproteomics, neuroprosthetics, etc.) can be seen by assessing the indexed, peer-reviewed literature of the past ten years [5]. As scientific knowledge and technological capabilities expand, and as such capabilities are possessed and exerted by a greater number of nations with evermore diverse cultures, the potential ethical, legal, and social issues that will arise – and their implications – become more complex, intricate, and challenging. Yet, as the speed of S/T progress and its translation into various aspects of medical diagnosis and treatment has increased, the formulation of guidelines, policies, and laws relevant to S/T research and use has slowed. Of course, guideline formulation and policy-making are deliberative processes, designed for careful consideration and durability of effect rather than for speed. It could be argued that this slowing of dictates guiding the expansive advancement and use of S/T innovation in clinical care reflect the complexity of novel developments, in that more time is required to accrue the information necessary to develop sound policies to prudently guide such research and its application(s). However, absent this consideration is the reality that S/T development and use is reliant, at least in part, upon guidelines and policies for support, sustenance, and direction. Thus, the dissonance between S/T advancement and policy formulation may result in much new medical S/T being unsupported, unguided, and/or unregulated.

### Ethics and biomedical science policy on the global stage

Knowledge about the process of politics is crucial as a variety of private entities (e.g., commercial businesses, venture capital investments) and political factions are exerting increasing influence on standards, regulations, and laws that bear upon the viability of the development and use of biomedical S/T advances as public good(s) [17]. In this regard, it is noteworthy that such S/T research – and applications in medicine – are no longer confined by national borders, and therefore, there is need to address not only nations’ needs, values, and mores, but the broader global dynamics that shape those “contexts in which ethical norms and delineations of human subjects are changing” [18], p.184. The constructs and dictates of western philosophy and ethics may no longer be considered as universally applicable or appropriate to prescribe the conduct of S/T research on the international scale, given that new biomedical S/T enterprises—and a global economic presence—of non-western nations, and commercial and cultural groups are on the rise. It is becoming clear that variance in the cultural values, traditions, and ethical standards between nations and regions can “… further entrench inequality, justifying some interventions while disallowing others” [18], p.184, and may prompt disequilibrium in the ways that research in and employment(s) of biomedical S/T advances are viewed, regarded, and engaged in global economies, politics, and culture(s).

This diversity necessitates more than a mere superficial appreciation, as there are risks of negatively bi-modal ethical consequences if socio-cultural considerations are less than thorough. On one hand there is the risk of assuming an exclusively western *ceteris paribus* stance and incurring frank ethical imperialism as western precepts and standards are enforced (or perhaps more accurately inflicted) upon other groups with dissimilar needs, values, and norms. On the other hand, there is the risk of treating any and all situations as *prima facie* contingencies, and defaulting to *laissez faire* ethical relativism. Both cases pose a potential risk for exertion of biopower and biopolitics [19]: the former via cultural dominance and/or suppression, and the latter through a form of locally constrictive, egoistic communitarianism that might foster “look the other way” practices among other nations and groups for the sake of ethical permissiveness and/or economic gain, and thereby permit what might be viewed as (internal) violations of essential goods and human rights [20]. It may be that some set of core precepts could serve as a common morality to undergird the conduct of biomedical S/T research and its use toward the provision of social good [21]. But, how would such “goods” be defined, and how are these definitions and the practices they instantiate to be developed, and decided upon, particularly when outcomes and products of S/T research – and the practice of medicine – are internationally and cross-culturally employed? To paraphrase philosopher Alasdair MacIntyre: what good, which justice, and whose rationalization [22]?

To be sure, any such bioethics must recognize cultural, if not some level of moral plurality [23]. At very least, some form of discursive ethics will be required to engage the type of casuistic analysis necessary to appreciate and navigate both socio-culturally-based moral variability, and how this affects – and might be affected by – the use of biomedical S/T advances upon the pluralist terrain of the 21^st^ century world stage [8]. If, in fact, the “new global shift” produces what has been colloquially described as a “flat world with creases” (i.e., highly accessible and interacting socio-economic cultural landscapes, punctuated by the emergence of new national powers and localized pockets of distinct power-identities of smaller nations, groups, and individuals in diaspora) then a more cosmopolitan bioethics that appreciates anthropological constants and variation(s), and is sensitive to standpoint, yet open to the dialectical formulation of moral constructs, standards, and directions will be needed [5,8,9,23-25].

### Toward a vision - and plan - forward

The goal will be to establish an educational system – if not paradigm – that will develop, enable, and empower actors upon the new world stage who will be able to direct the use of S/T innovation through an understanding of both science, and “…principles which represent the full moral experience and concerns of real human beings” [26], p.18. In many ways, we believe that this directly addresses 1) concerns about the widening schism between science and the humanities [27], 2) the ongoing debate about the validity and value of ethical expertise in directing the scope and tenor of science and technology (see, for example Selinger and Crease 2006 [28], for overview), and 3) a tendency toward overt neo-scientism, technophilia and/or technocentricism that fails to appreciate “real world” circumstances of human ecology and culture that are affected by—and concomitantly affect—the conduct, use, and potential misuse of science, technology, and medicine [29-32].

In overview, this integrated system would progressively weave historicity, ethics, and policy studies into STEM courses over the course of students’ educational career. Early on, at the high school level, course content-specific examples could and should be integrated into regular class work. High school level curricula are generally more prescribed than those at higher levels of education and as such, integration would best be facilitated through collaborative initiatives connecting STEM teachers with those in the humanities. An increase in grant funding from organizations such as NSF and NIH, or through university outreach opportunities, would provide substantial support for such initiatives, particularly since individual grants at this educational level are often smaller in amount.

At the undergraduate, graduate, and medical school levels, an *In-STEPS* curriculum could be supported in a number of different ways. University funding should support representatives of academic departments collaborating toward both the integration of ELSI into STEM courses and STEM applications into classes in the humanities. Similarly, ELSI should be integrated into medical school curricula as an expansion of the current ethical coursework and training. Increased grants from NSF and NIH in scientific ethics education—as an expansion of funding programs that support the current RCR initiatives—should support the development of new interdisciplinary seminars group-taught by professors in multiple departments and schools so as to guide study of the reciprocity of STEM and ELSI; such interdisciplinary courses could also access funding sources in the humanities, thereby enhancing overall fiscal support as well. Combined funding initiatives that encourage collaboration between humanities and STEM-related organizations would also facilitate integration in the professional realm and encourage the bilateral translation of academic coursework to the professional scenarios.

The effect(s) of such an *In-STEPS* paradigm extend beyond the educational sphere to impact and encompass the scope and tenor of interdisciplinary professional development. Working within the *In-STEPS* approach, each field would foster and encompass its own particular means of integrating related STEM-ELSI issues. Opportunities for trans-professional collaboration could be supported through intra- and inter-organizational networking opportunities. By developing and sustaining cross-professional relationships, consideration, and communication between medicine, the sciences, engineering, and the humanities can be focused both upon understanding how science and technology offers—and provides—potential to improve the human condition, as well as upon acknowledging and bearing the responsibility to define, prevent, and/or mitigate the ethical, legal, and social burdens, risks, and harms that such S/T advances might yield upon the current and future world stage.

## Summary

Just as research is fostered, supported, and sustained through the structure and function of the “triple helix” conjoining academia, government, and private industry, so too must be the educational training of future biomedical professionals who will bear responsibility for the effects that an ever-expanding fund of scientific and biotechnological knowledge and capability will yield upon the practice of medicine, humanity, world culture, and global environments, writ both small and large. These professionals must put to use their integrated knowledge of the ethical, legal, and social issues and how they work within and inform the continued development and application of scientific and technological advances in the medical diagnoses and care. The model we propose is specifically aimed at addressing this need, and our ongoing work is dedicated to collaborating with multidisciplinary, international colleagues and institutions to further develop and realize the *In-STEPS* paradigm to affect a more scientifically capable, yet sustainably humanitarian, ethos and *telos* of medicine.

## Competing interests

The authors declare that they have no competing interests.

## Authors’ contributions

MA conducted primary research and drafted the manuscript. JG developed the core constructs, advised research and helped to draft the manuscript. Both authors read and approved the final manuscript.

## Authors’ information

At the time of this work, MA was a graduate student at Georgetown University and Visiting Scholar at the Potomac Institute for Policy Studies. MA currently serves as a Research Analyst at the Presidential Commission for the Study of Bioethical Issues, however, this article was written in her private capacity. No official support or endorsement by the Presidential Commission for the Study of Bioethical Issues or the Department of Health and Human Services is intended, nor should be inferred.

JG is Chief of the Neuroethics Studies Program at the Edmund D. Pellegrino Center for Clinical Bioethics, and Professor in the Division of Integrative Physiology, Department of Biochemistry, and Graduate Liberal Studies Program of Georgetown University, Washington, DC. He is Clark Fellow in Neurosciences and Ethics at the Human Science Center of the Ludwig-Maximilians Universität, Munich, Germany; William H. and Ruth Crane Schaefer Distinguished Visiting Professor of Neuroscience and Ethics at Gallaudet University, Washington, DC, USA, and a Senior Fellow of the Potomac Institute for Policy Studies, Washington, DC, USA.

## Pre-publication history

The pre-publication history for this paper can be accessed here:

http://www.biomedcentral.com/1472-6920/13/58/prepub

## References

[B1] Giordano J Hutchison P Benedikter R Regrounding medicine amidst a technological imperative and Post-Modern mindset Int J Polit Cult Soc 2010 10 10 10.1186/1747-5341-5-17

[B2] Pellegrino ED Thomasma DC A Philosophical Basis of Medical Practice: Toward a Philosophy and Ethic of Healing Professions 1981 New York: Oxford University Press

[B3] Benedikter R Giordano J The outer and inner transformation of the global sphere through technology: the state of two fields in transition New Global Stud 2011 5 2 10.2202/1940-0004.1129

[B4] Benedikter R Giordano J FitzGerald K The future of the self-image of the human being in the age of transhumanism, neurotechnology and global transition J Futures 2010 42 10 1102 1109 10.1016/j.futures.2010.08.010

[B5] Giordano J Giordano J Neurotechnology as a demiurgical force: Avoiding Icarus’ Folly Neurotechnology: Premises, Potential and Problems 2012 Boca Raton: CRC Press 1 14

[B6] McMillan GS Narin F Deeds DL An analysis of the critical role of public science in innovation: the case of biotechnology Res Policy 2000 29 1 8 10.1016/S0048-7333(99)00030-X

[B7] Wurzman R Inter-disciplinarity and constructs for STEM education: at the edge of the rabbit hole Synesis: a Journal of Science, Technology, Ethics and Policy 2010 1 32 35

[B8] Giordano J NATO Science for Peace and Security Series, Ashok V, Braman E, Sussman P Integrative convergence in neuroscience: trajectories, problems and the need for a progressive neurobioethics Technological Innovation in Sensing and Detecting Chemical, Biological, Radiological, Nuclear Threats and Ecological Terrorism 2012 New York: Springer 233 265

[B9] Giordano J Keeping science and technology education In-STEP with the realities of the world stage: Inculcating responsibility for the power of STEM Synesis: a Journal of Science, Technology, Ethics and Policy 2012 3 1 5

[B10] Chamany K Allen D Tanner K Making biology learning relevant to students: integrating people, history, and context into college biology teaching CBE Life Sci Educ 2008 7 267 278 10.1187/cbe.08-06-0029 18765745 PMC2527976

[B11] Garrett JM Bird SJ Ethical issues in communicating science Sci Eng Ethics 2000 6 435 442 10.1007/s11948-000-0001-7 11228768

[B12] Garrett JM Triman KL Resources and strategies to integrate the study of ethical, legal, and social implications of genetics into the undergraduate curriculum Adv Genet 2009 66 35 59 19737637 10.1016/S0065-2660(09)66002-8

[B13] National Institutes of Health (NIH) Update on the requirement for instruction in the responsible conduct of research http://grants1.nih.gov/grants/guide/notice-files/NOT-OD-10-019.html

[B14] National Science Foundation (NSF) Responsible conduct of research (RCR) http://www.nsf.gov/bfa/dias/policy/rcr.jsp

[B15] Booth JM Garrett JM Instructors’ practices in and attitudes toward teaching ethics in the genetics classroom. Edited by P. Pukkila Genetics 2004 168 1111 1117 10.1534/genetics.103.023077 15579673 PMC1448769

[B16] Eckles RE Meslin EM Gaffney M Helft P Medical ethics education: Where are we? Where should we be going? A review Acad Med 2005 80 12 1142 1152 10.1097/00001888-200512000-00019 16306292

[B17] Barley SR Corporations, democracy, and the public good J Manage Inquiry 2007 16 3 201 215 10.1177/1056492607305891

[B18] Petryna A Ethical variability: drug development and globalizing clinical trials Am Ethinol 2005 32 2 183 197 10.1525/ae.2005.32.2.183

[B19] Foucault M The Birth of the Clinic: An Archaeology of Medical Perception 1994 New York: Vintage Press

[B20] Anderson MA Fitz N Howlader D Giordano J Neurotechnology research and the world stage: Ethics, biopower and policy Neurotechnology: Premises, Potential and Problems 2012 Boca Raton: CRC Press 287 300

[B21] Gert B Morality: Its Nature and Justification 2005 New York: Oxford University Press

[B22] MacIntyre A Whose Justice? Which Rationality? 1988 Notre Dame: University of Notre Dame Press

[B23] Engelhardt HT Bioethics in the third millennium: some critical anticipations Kennedy Inst Ethic J 1999 9 3 225 243 10.1353/ken.1999.0018 11657716

[B24] Giordano J Benedikter R An early - and necessary - flight of the owl of Minerva: neuroscience, neurotechnology, human socio-cultural boundaries, and the importance of neuroethics J Evolution Technol 2012 22 1 14 25

[B25] Wallace KA Common morality and moral reform Theor Med Bioeth 2009 30 55 68 10.1007/s11017-009-9096-2 19205923

[B26] Widdows H Western and Eastern principles and globalised bioethics Asian Bioethics Review 2011 3 1 14 22

[B27] Snow CP The Two Cultures 1959 Cambridge: Cambridge University Press

[B28] Selinger E Crease RP The Philosophy of Expertise 2006 New York: Columbia University Press

[B29] Ballard EG Man and Technology: Toward the Measurement of a Culture 1978 Pittsburgh: Duquesne University Press

[B30] Borgmann A Technology and the Character of Contemporary Life: A Philosophical Inquiry 1984 Chicago: University of Chicago Press

[B31] Jonas H Das Prinzip Verantwortung: Versuch einer Ethik für die technologische Zivilisation. 1979 Frankfurt am: Insel Verlg

[B32] Lenk H Technokratie als Idelogie: Sozialphilosophische Beitrage zu einem politischen Dilemma 1973 Stuttgart: Kohlhammer Verlaf

